# Differential MicroRNA Expression of miR-21 and miR-155 within Oral Cancer Extracellular Vesicles in Response to Melatonin

**DOI:** 10.3390/dj7020048

**Published:** 2019-05-01

**Authors:** Matthew Hunsaker, Greta Barba, Karl Kingsley, Katherine M. Howard

**Affiliations:** 1 Department of Clinical Sciences, School of Dental Medicine, University of Nevada, Las Vegas, 1700 W. Charleston Blvd., Las Vegas, NV 89106, USA; matthew.hunsaker@sdm.unlv.edu (M.H.); greta.barba@sdm.unlv.edu (G.B.); 2 Department of Biomedical Sciences, University of Nevada, Las Vegas—School of Dental Medicine, 1001 Shadow Lane, Las Vegas, NV 89106, USA; katherine.howard@unlv.edu

**Keywords:** microRNA, oral cancer, melatonin

## Abstract

Objective: Extracellular vesicles derived from oral cancer cells, which include Exosomes and Oncosomes, are membranous vesicles secreted into the surrounding extracellular environment. These extracellular vesicles can regulate and modulate oral squamous cell carcinoma (OSCC) progression through the horizontal transfer of bioactive molecules including proteins, lipids and microRNA (miRNA). The primary objective of this study was to examine the potential to isolate and evaluate extracellular vesicles (including exosomes) from various oral cancer cell lines and to explore potential differences in miRNA content. Methods: The OSCC cell lines SCC9, SCC25 and CAL27 were cultured in DMEM containing 10% exosome-free fetal bovine serum. Cell-culture conditioned media was collected for exosome and extracellular vesicle isolation after 72 h. Isolation was completed using the Total Exosome Isolation reagent (Invitrogen) and extracellular vesicle RNA was purified using the Total Exosome RNA isolation kit (Invitrogen). Extracellular vesicle miRNA content was evaluated using primers specific for miR-16, -21, -133a and -155. Results: Extracellular vesicles were successfully isolated from all three OSCC cell lines and total extracellular vesicle RNA was isolated. Molecular screening using primers specific for several miRNA revealed differential baseline expression among the different cell lines. The addition of melatonin significantly reduced the expression of miR-155 in all of the OSCC extracellular vesicles. However, miR-21 was significantly increased in each of the three OSCC isolates. No significant changes in miR-133a expression were observed under melatonin administration. Conclusions: Although many studies have documented changes in gene expression among various cancers under melatonin administration, few studies have evaluated these effects on microRNAs. These results may be among the first to evaluate the effects of melatonin on microRNA expression in oral cancers, which suggests the differential modulation of specific microRNAs, such as miR-21, miR-133a and miR-155, may be of significant importance when evaluating the mechanisms and pathways involved in melatonin-associated anti-tumor effects.

## 1. Introduction

Many health benefits have been associated with melatonin expression and in more recent years, melatonin supplementation—including effects on sleep regulation and modulation in various states of health and disease [1,2,3]. Additional studies have demonstrated that melatonin may have beneficial effects on other conditions ranging from cardiovascular physiology, malarial disease, and neuropsychological conditions and delirium prevention [4,5,6,7]. These studies have prompted greater examinations of the potential for melatonin and other complementary and alternative therapies to integrate with cancer therapy and treatment [8,9].

Due to the nature, structure and function of melatonin, much of this research has focused on hormone-dependent tumors, such as breast and pancreatic cancers [10,11,12]. However, the preventive and therapeutic actions of melatonin have also been observed in many other types of tumors, including oral and esophageal cancers [13,14,15,16,17]. Many of these effects have been associated with the potential for transcriptional modulation of genes important to oncogenesis and cancer progression, including apoptosis, proliferation and angiogenesis [18,19,20]. 

More recent studies have demonstrated that melatonin may also modulate the expression of non-coding microRNAs (or, miRNA), which modulate many aspects of tumor phenotypes in breast, liver and gastric cancers [21,22,23]. In fact, melatonin may modulate expression of specific microRNAs that function across multiple tumor types, such as miR-155, miR-133a and miR-21 [24,25,26]. 

Although previous studies from this group have evaluated changes to oral cancer gene expression under melatonin administration, as well as differential expression of oral cancer exosomes, extracellular vesicles, and microRNAs, no studies to date have analyzed the effects of melatonin on microRNA expression in oral cancers [27,28]. Based upon the paucity of evidence in this area, the primary goal of this project was to evaluate the effects of melatonin administration on the expression of specific microRNAs, such as miR-21, miR-133a and miR-155 among oral cancers. 

## 2. Methods

### 2.1. Tissue Culture

Three human oral squamous cell carcinoma cell lines were obtained from American Tissue Type Culture Collection (Manassas, VA, USA): SCC9 (CRL-1629), SCC25 (CRL-1628) and CAL27 (CRL-2095). Cell cultures were maintained in Dulbecco’s Modified Eagle’s Medium (DMEM) from Hyclone (Logan, UT, USA) using the formulation with 4.0 mM l-glutuamine, 4.5 g/L glucose and 110 mg/L sodium pyruvate. DMEM was further supplemented with streptomycin (100 μg/mL) and penicillin (100 U/mL) and 10% fetal bovine serum (FBS) also obtained from Hyclone. Cell cultures were maintained in BD Falcon (Bedford, MA, USA) tissue culture treated flasks in humidified tissue culture incubators with 5% CO_2_ at 37 °C. 

### 2.2. Reagents

Melatonin (*N*-acetyl-5-methoxy-tryptamine) used in this study was purchased from Thermo Fisher Scientific (Fair Lawn, NJ, USA). Each experiment was performed with and without the addition of melatonin to the cell culture medium at 10 μg/mL for 72 h, as previously described [28]. This concentration was selected to approximate serum concentrations of melatonin found in serum and saliva following administration as an over-the-counter supplement [28,29,30]. Non-treated cells were used as the negative control and each experiment was repeated in triplicate (n = 3) for each cell line and both conditions (negative control, experimental).

### 2.3. Intact Exosome Isolation

Cell cultures were transferred into DMEM containing exosome-depleted FBS (with and without the addition of melatonin) for the 24 h before the isolation of exosomes. In order to remove any cells or cellular debris, the conditioned medium was removed from each tissue culture flask and centrifuged at 2000× *g* for 30 min. The supernatant was then extracted and combined with Total Exosome Isolation reagent from Life Technology (Waltham, MA) and refrigerated overnight according to the manufacturer protocol. Extracellular vesicles were then isolated by centrifugation at 10,000× *g* at 4 °C for one hour. The exosome-containing pellets were resuspended in 200 μL of Phosphate Buffered Saline (PBS) solution. 

### 2.4. RNA Extraction from Exosomes

An equal volume of 2X Denaturing solution from Life Technology was added to the exosome resuspension and incubated on ice for five minutes. One volume of Phenol:Chloroform was added to the solution and centrifuged at 10× *g* at 4 °C for five minutes. The upper (aqueous) phase was removed and 1.25 volumes of ethanol (EtOH) were then added. The sample was then transferred into a filter and centrifuged at 10× *g* for 15 s. Each sample/filter was then washed using miRNA Wash Solution 1 and centrifuged at 10× *g* for 15 s before repeating this process with Wash Solution 2/3. To remove any residual fluid, each sample/filter was then centrifuged for 15 additional seconds. Each filter was then placed into a new collection tube and 100 μL of heated RNase water was applied prior to centrifugation for 30 s. The exosome RNA was contained in the collected flow through. 

### 2.5. TaqMan microRNA Assays

Reverse transcription was accomplished using 15 L reactions that consisted of 10X Reverse Transcription Buffer, RNase inhibitor, 100 mM deoxyribonucleotide triphosphate (dNTP) and MultiScribe Reverse Transcriptase containing 3 L of miR specific primer. Thermal cycler settings were 16 °C for 30 min, 42 °C for 30 min, 85 °C for five minutes, followed by cooling to 4 °C. 

Quantitative polymerase chain reaction (qPCR) was accomplished in 20 μL reactions using the TaqMan Small RNA assay, TaqMan Universal PCR Master Mix II and corresponding product from the reverse transcription reaction. Thermal cycler settings were 50 °C for two minutes, 95 °C for 10 min, then 40 cycles of 95 °C for 15 s and 60 °C for 60 s. Standard curves were made doing a five-fold serial dilution of cDNA for miR-16, the endogenous reference gene (positive control) for exosomal miRNA.

### 2.6. Statistical Analysis

Two-tailed t-tests were used to assess any statistical differences between the relative quantity of microRNAs (miR-21, miR-133a, miR-155) isolated from each cell line under control and experimental conditions. Histograms of qPCR expression are reported, including standard deviation (SD). Statistical significance was set at an alpha level, *α* = 0.05. 

## 3. Results

Each of the three oral cancer cell lines generated visible exosome precipitation pellets from both the control and experimental assays, which were then processed to extract exosome RNA for quantitative PCR. The TaqMan MicroRNA assays for miR-21, miR-133a and miR-155 were performed and standard curves generated from cDNAs, thus allowing for relative quantitation of each of the target microRNAs. 

**Figure 1:**
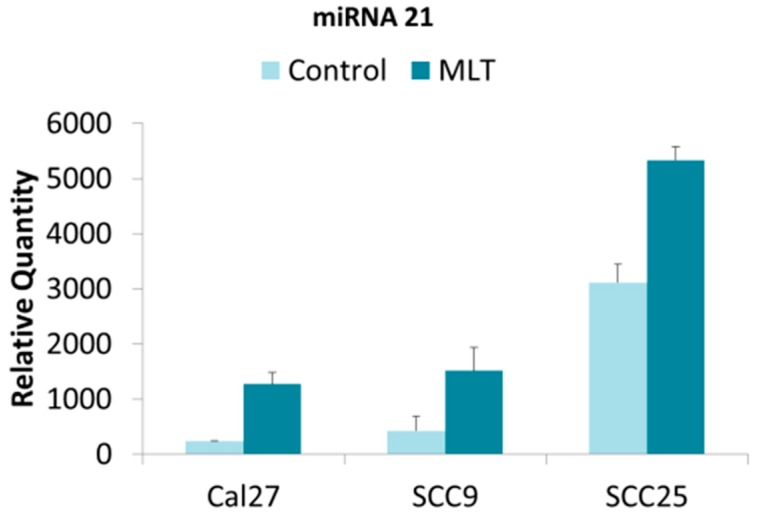
Relative miR-21 expression in oral cancers under melatonin (MLT) administration. Histogram of quantitative PCR from cDNAs derived from extracellular vesicles demonstrated differential baseline expression of miR-21 including standard deviation (SD), which was significantly increased in all cell lines under melatonin administration, *p* < 0.05 (CAL27, *p* < 0.001; SCC9, *p* < 0.001; SCC25, *p* < 0.001).

The results for miR-21 demonstrated that all three oral cancer cell lines exhibited miR-21 expression in the extracellular vesicle isolates, although the relative expression of miR-21 varied considerably (Figure 1). For example, the relative quantity of miR-21 in extracellular vesicles isolated from CAL27 and SCC9 in the negative controls was similar, but significantly lower than was observed in SCC25 cells. The addition of melatonin induced significant increases in the relative quantity of miR-21 in extracellular vesicles from all three cell lines (CAL27, SCC9, SCC25)—regardless of the baseline expression observed (*p* < 0.05). 

**Figure 2:**
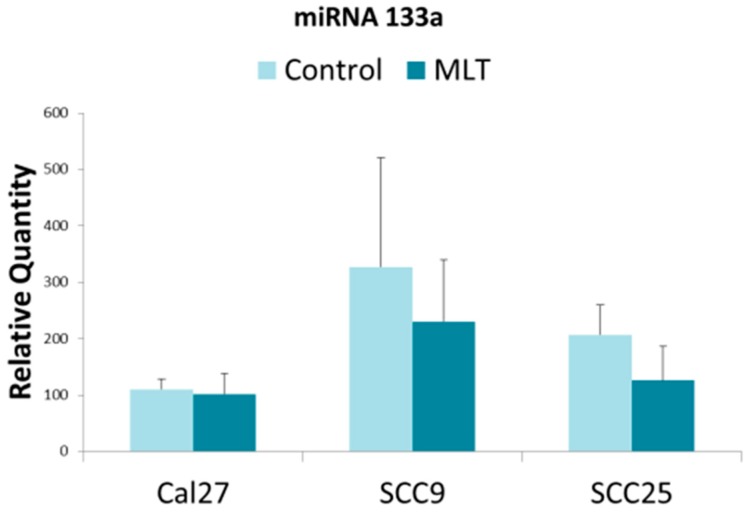
Relative miR-133a expression in oral cancers under melatonin (MLT) administration. Histogram of quantitative PCR from cDNAs derived from extracellular vesicles demonstrated differential baseline expression of miR-133a including standard deviation (SD), which was not significantly altered in any cell line under melatonin administration (CAL27, *p* = 0.11; SCC9, *p* = 0.634; SCC25, *p* = 0.411).

The results for miR-133a also demonstrated that each of the oral cancer cell lines harbored miR-133a within their respective extracellular vesicles, with differential expression also observed (Figure 2). For example, the relative quantity of miR-133a in extracellular vesicles isolated from CAL27 was significantly lower than that observed from either SCC9 or SCC25—although the greatest relative quantity was observed from SCC9. However, the addition of melatonin did not have any significant or observable effect on the relative quantity of miR-133a in extracellular vesicles from any of the oral cancer cell lines in this study (CAL27, *p* = 0.11; SCC9, *p* = 0.634; SCC25, *p* = 0.411). 

**Figure 3:**
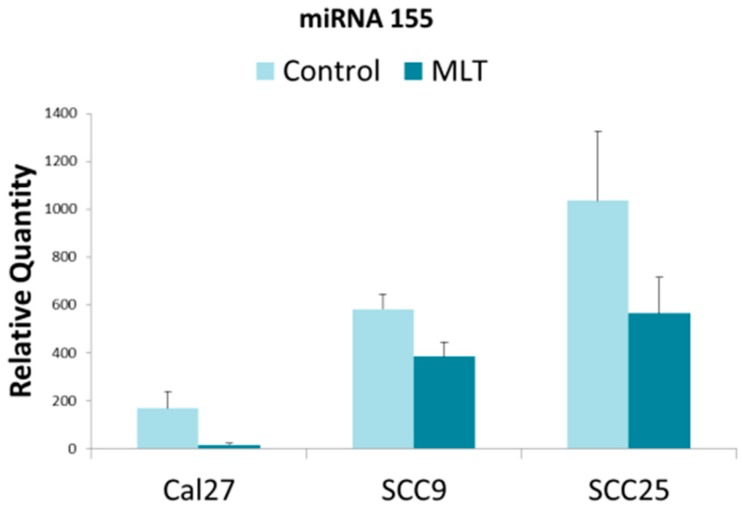
Relative miR-155 expression in oral cancers under melatonin (MLT) administration. Histogram of quantitative PCR from cDNAs derived from extracellular vesicles demonstrated differential baseline expression of miR-155 including standard deviation (SD). Administration of melatonin significantly reduced expression of miR-155 in all three cell lines, *p* < 0.05 (CAL27, *p* < 0.001; SCC9, *p* = 0.003; SCC25, *p* = 0.039).

Finally, the results for miR-155 confirmed each of the oral cancer cell lines contained miR-155 in the extracellular vesicles, revealing that each cell line also exhibited differential expression (Figure 3). The lowest level of baseline miR-155 expression was observed in CAL27, with relatively higher levels observed in SCC9 and even greater expression among SCC25 cells. Interestingly, the addition of melatonin significantly decreased miR-155 expression in all three cell lines, *p* < 0.05 (CAL27, *p* < 0.001; SCC9, *p* = 0.003; SCC25, *p* = 0.039).

**Figure 4:**
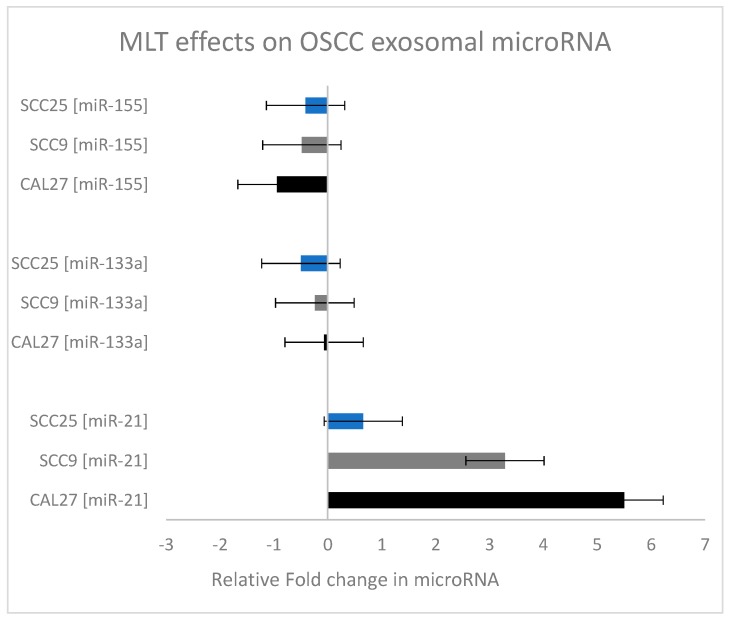
Relative fold change in microRNA expression in oral cancers under melatonin (MLT) administration. Calculation of the relative fold change in miRNA expression (experimental/baseline control) revealed significant increases in miR-21, with relatively little change to miR-133a and some inhibition of miR-155 (particularly in the CAL27 cells).

To provide a comparison of the effects of melatonin on these microRNAs, the relative fold change in microRNA expression was plotted for each microRNA and oral cancer cell line (Figure 4). These data clearly reveal the inhibitory effect of melatonin on miR-155 in all three oral cancer cell lines. In addition, the significant increase in miR-21 expression following melatonin administration is demonstrated—with the greatest effects observed among the SCC9 and CAL27 cell lines. 

Finally, to evaluate any effects of melatonin administration on the endogenous control, levels miR-16 were also evaluated (Table 1). These data suggest no significant differences in miR-16 expression between treated and untreated cells from this experiment. 

## 4. Discussion

Although previous studies from this group have evaluated changes to oral cancer gene expression under melatonin administration (as well as differential expression of oral cancer extracellular vesicles, exosomes, and microRNAs) no studies to date have analyzed the effects of melatonin on microRNA expression in oral cancers [27,28]. This study was able to successfully evaluate the effects of melatonin administration on the expression of specific microRNAs, such as miR-21, miR-133a and miR-155 among specific oral cancer cell lines, including CAL27, SCC9 and SCC25. These data clearly demonstrated differential responses between each cell line, as well as between each specific microRNA.

For example, the expression of miR-21 has been demonstrated to be a useful biomarker and prognostic tool, which is strongly correlated with decreased survival [31,32]. This specific microRNA has been proposed in several independent studies as one of the critical factors that might be involved with oral squamous cell carcinoma progression [33,34,35]. Although melatonin has been associated with tumor inhibition in other studies [8,9,10], this study is the first to assess the effect of melatonin on miR-21 in exosome and extracellular vesicle RNA from oral cancers. One potential explanation for the surprising observations in this study that demonstrated increased miR-21 expression following melatonin administration relates to the variety of transcription factor binding sites in the miR-21 promoter regions, including AP1, STAT3 and many others [36,37]. It is possible that many other transcriptional mediators, such as melatonin, may also facilitate miR-21 transcription—although no temporal (or longer-term) information was gathered from this study to determine if miR-21 expression remains stable under melatonin supplementation. 

The inhibition of miR-155 under melatonin administration was significant, as miR-155 is known to modulate additional pathways of cellular proliferation and apoptosis in oral cancers through p27Kip1 and BCL6/cyclin D2 [38,39]. That melatonin administration may be associated with down-regulation of miR-155 expression may appear to support previous observations that miR-155 is an important regulator of oral squamous cell carcinoma metastasis and is associated with poor prognosis [40,41]. In fact, recent evidence has suggested that miR-155 may be a critical transcriptional activator and prognostic biomarker that may be sufficient to induce changes to oral cancer proliferation [42]. One previous study did report that melatonin administration inhibited proliferation and invasion of glioma cells through transcriptional repression of miR-155, although no other study to date has evaluated these effects among oral cancers [24]. 

It important to note that these may be the first published evidence of these effects among oral cancers and will need to be further strengthened by other studies that include additional oral cancer cell lines, as well as primary tumor isolates. It would also be important to note that the study limitations did not allow for longer term, temporal analysis of melatonin-induced changes to these cellular phenotypes—which will be an important component of future studies from this group. However, previous studies from this group have identified reductions in cellular viability in response to melatonin, which may be important for contextualizing these results [43,44]. Finally, there are many additional microRNAs that may be important mediators of clinical outcomes and cellular phenotypes—therefore, future studies should also evaluate any potential effects of melatonin on these microRNAs [45,46]. 

## 5. Conclusions

Although many studies have documented changes in gene expression among various cancers under melatonin administration, few studies have evaluated these effects on microRNAs. These results may be among the first to evaluate the effects of melatonin on microRNA expression in oral cancers, which suggests the differential modulation of specific microRNAs, such as miR-21, and miR-155, may be of significant importance when evaluating the mechanisms and pathways involved in melatonin-associated anti-tumor effects.

## Figures and Tables

**Table 1:** Analysis of miR-16 expression.

Cell Type	Control miR-16 C_T_	Melatonin miR-16 C_T_
SCC9	29.3 ± 0.23	29.5 ± 0.27
SCC25	30.3 ± 0.11	29.9 ± 0.32
Cal27	35.6 ± 0.31	35.7 ± 0.24
